# Biological Nanoscaffolds from Hierarchical Construction to Applications

**DOI:** 10.3390/molecules31050812

**Published:** 2026-02-28

**Authors:** Yicong Zhang, Haolu Shi, Yijia Li, Yanlin Shen, Tingting Wang, Junqiu Liu

**Affiliations:** Key Laboratory of Organosilicon Chemistry and Material Technology, Ministry of Education, College of Material, Chemistry and Chemical Engineering, Hangzhou Normal University, Hangzhou 311121, China; yicongzhangzoe@163.com (Y.Z.); haolushihznu@163.com (H.S.); tianrz17@163.com (Y.L.); syl923225575@yeah.net (Y.S.)

**Keywords:** biological nanoscaffold, nucleic acid scaffold, protein scaffold, protein assembly, protein cage, biomimetic

## Abstract

Inspired by natural scaffolds, artificial scaffolds have garnered significant attention in recent years. Compared with synthetic scaffolds such as organic and polymer scaffolds, biological scaffolds from the foundational biomolecules nucleic acids (DNA/RNA) and proteins demonstrate distinct advantages in the assembly of inorganic nanoparticles and proteins, as well as in drug delivery. These advantages stem from their exquisite spatial structures, genetically encoded programmability, and their favorable biocompatibility, which is attributed to natural building blocks and degradable backbones that minimize long-term cytotoxicity. The intrinsic properties and structural symmetry of biomacromolecules as building blocks often determine the properties of the corresponding assemblies, and thus greatly influence their functions. In this review, we classify bottom-up constructed biological scaffolds according to these two primary constituent classes (nucleic acids and proteins) to examine their framework structures and key features. We also discuss the relevant applications of artificial bioscaffolds. As an emerging class of nanomaterial with precise structures and genetic programmability, biological scaffolds hold significant promise for future development.

## 1. Introduction

Nanoscaffold structures are integral to nature due to their involvement in many natural life activities, such as cellular reactions, metabolic pathways, and signal transduction [1,2]. For example, natural proteins spontaneously assemble into highly organized multi-enzyme complexes that drive various metabolic reactions. Moreover, scaffold structures serve as the fundamental basis for the anchoring of substances, delivery of biomolecules, and construction of biological nanostructures. The wisdom of nature has provided great inspiration for the “bottom-up” design of a new generation of template structures [3]. Natural macromolecules, such as proteins and nucleic acids, are scaffold structures with inherent catalytic and informational activity, and they can directly inspire the design of man-made scaffolds. However, recapitulating the sophistication and function of natural assemblies with synthetic components presents a fundamental challenge in de novo design and scalable fabrication.

Scaffolds can be divided into synthetic scaffolds and natural biological scaffolds according to their composition. To date, many hierarchically ordered nanoscaffolds have been synthesized to better explore the mysteries of nature, such as metal–organic frameworks (MOFs), covalent organic frameworks (COFs), hydrogen-bonded organic frameworks (HOFs), and biomolecule-based scaffolds (including nanowires, nanotubes, nanoparticles, and other nanostructures) [4,5,6,7,8,9]. Compared with many synthetic scaffolds, bioscaffolds are constructed from natural biomolecules through genetic engineering or chemical modifications. This biomimetic foundation can offer a favorable starting point for achieving biocompatibility, as they inherently possess motifs recognizable by biological systems. However, their actual biocompatibility profile—encompassing immunogenicity, clearance kinetics, and toxicity—is not intrinsic but must be actively engineered and validated. It is critically dependent on specific design choices, such as the introduction of stealth coatings, the control of surface charge and hydrophilicity, and the administered dose [10]. Furthermore, performance varies significantly across different biological contexts and target tissues [11]. This underscores a broader theme: the ultimate utility of any engineered bioscaffold is contingent upon overcoming a series of interconnected challenges spanning from precise synthesis and functionalization to stability in complex biological milieus.

**Scheme 1:**
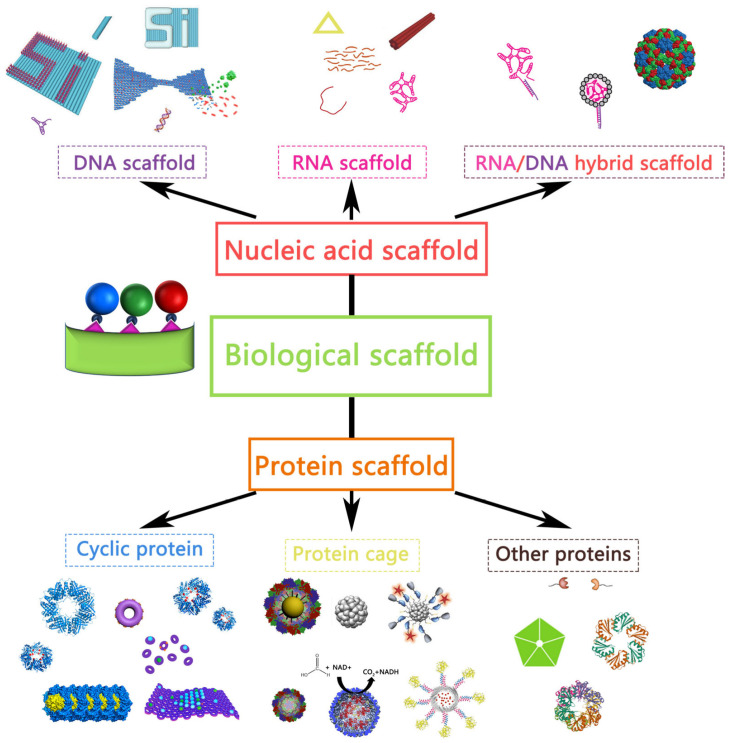
Biological scaffolds based on distinct classes of biomacromolecules: nucleic acid-based scaffolds (DNA, RNA and hybrid-paired structures) and protein-based scaffolds. (Classified by the structural features of their building blocks).

Bioscaffolds are nanostructures with specific biological functions constructed from biomolecules, which are generally composed of nucleic acids and proteins. Artificial biological scaffolds are considered to have great biomimetic potential because of their building blocks and structural features that resemble those of natural scaffolds. Nucleic acid scaffolds are formed by intermolecular hydrogen bonds formed between single-stranded nucleic acid chains according to strict Watson–Crick base-pairing rules, while protein scaffolds are typically assembled via classical protein self-assembly strategies [12]. Among them, ring- or cage-shaped protein assemblies exhibit advantages for the construction of biological scaffolds due to their distinctive architectures, such as a large editable outer surface and stable internal cavity. Furthermore, biological scaffolds tend to have relatively ordered molecular arrangements and spatial structures at the nanoscale, which allows them to anchor biomolecules in an orderly manner. On the other hand, both nucleic acids and proteins, whose sequences are encoded in DNA and translated into functional forms, can be rationally designed and modified using genetic engineering, enabling precise editing of the building blocks of biological scaffolds at the source and thereby facilitating the development of nano-templates [3]. Following the wisdom of nature, many nanoscaffolds based on natural biomolecules, such as nucleic acids and proteins, have been successfully constructed and widely employed (Scheme 1) [13,14]. Translating this precise molecular-level control into equally predictable and robust macroscopic assembly, however, remains a non-trivial endeavor.

In fact, the construction of biological scaffolds by assembling biomolecules is an important research area in nanoscience. Rational design and control of biomolecules have been regarded as a classic bottom-up strategy for constructing biological scaffolds. The structure and intrinsic properties of the biomolecules as building blocks usually have a significant impact on the characteristics and the subsequent functions of the scaffolds they form. This review aims to provide a design-rule oriented compendium and a structured mapping of biological nanoscaffolds by systematically classifying them based on their fundamental constituent units—nucleic acids and proteins. Accordingly, the review is organized into dedicated sections that first examine nucleic acid-based scaffolds (DNA and RNA) and then protein-based scaffolds, elucidating how the inherent properties of these biomolecules dictate assembly logic, structural features, and functional capabilities, while extracting cross-platform design principles and strategic trade-offs applicable across scaffold systems. Furthermore, we establish explicit correlations between specific scaffold architectures and their performance in targeted applications such as multi-enzyme catalysis, targeted drug delivery, and biosensing, offering a practical roadmap for matching scaffold design to functional requirements. Finally, beyond highlighting achievements in programmable geometry and function, we provide a balanced perspective that critically examines major translational hurdles—spanning synthesis, functionalization, stability, and regulatory pathways—to present a realistic assessment of the field’s current state and future trajectory.

## 2. Nucleic Acid Scaffolds

With the development of nucleic acid nanotechnology and computational modeling techniques, the construction of DNA- or RNA-based nanostructures has advanced significantly [14,15]. As one of the most important components of biological scaffolds, the nucleic acid-based architectures have evolved from one-dimensional structures to two-or three-dimensional complex structures through DNA/RNA origami, owing to their programmability and structural predictability, and hold promise for diverse applications in nanotechnology, biology, physics, and medicine [10].

### 2.1. DNA Scaffolds

The advent of DNA origami, a key branch of DNA nanotechnology, has provided a powerful and programmable platform for constructing intricate two- and three-dimensional nanostructures through bottom-up self-assembly [16,17]. This capability has significantly expanded the utility of DNA scaffolds across multiple disciplines, most notably in the precise assembly of inorganic nanomaterials, the development of sophisticated drug delivery systems, and the controlled organization of proteins and enzymes.

**Figure 1:**
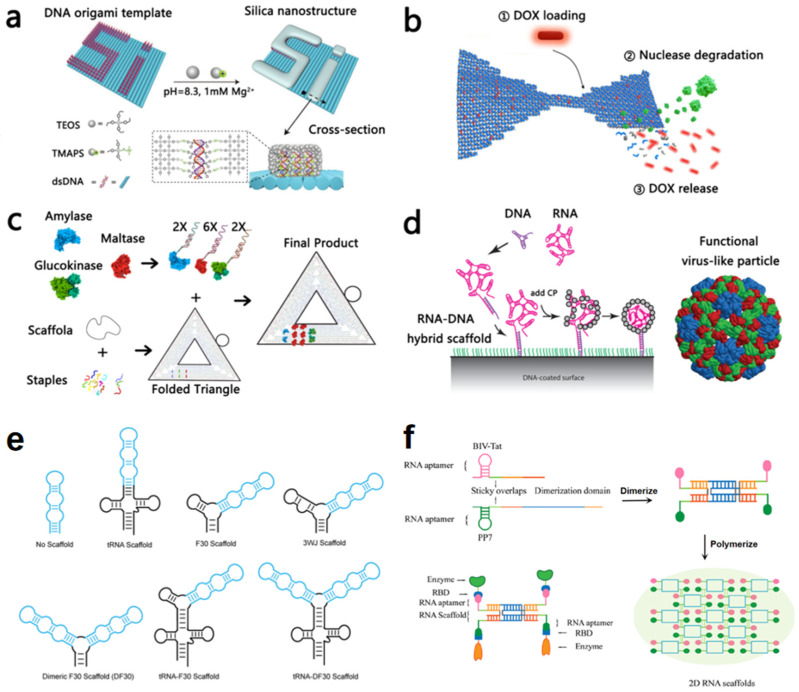
Nucleic acid scaffolds. (**a**) Schematic illustration of site-specific synthesis of silica nanostructures on a DNA origami template [26]. Copyright 2020 Wiley. (**b**) Schematic of the doxorubicin (DOX) loading into a DNA origami nanostructure (DON) and subsequent release upon enzymatic degradation [28]. Copyright 2021 Nucleic Acids Research. (**c**) Schematic of the DNA–enzyme assembly workflow from enzyme expression to scaffolded positioning on a DNA origami triangle [54]. Copyright 2019 American Chemical Society. (**d**) Schematic showing the assembly of VLPs on a DNA-functionalized (green strands) surface [83]. Copyright 2015 American Chemical Society. (**e**) Design of Pepper (blue) in different RNA scaffolds (black) [70]. Copyright 2022 American Chemical Society. (**f**) Two RNA oligonucleotides were united to form 2D scaffolds through dimerization and polymerization of the sticky end base pairs [71]. Copyright 2024 American Chemical Society.

For protein and enzyme science, DNA scaffolds provide a versatile means to organize biomolecules with nanometer precision. In inorganic materials science, DNA scaffolds offer unprecedented control over the spatial arrangement of nanoparticles, which is fundamental to tuning their collective optical, electronic, and catalytic properties [18]. This capability is particularly valuable for plasmonic materials such as gold and silver, whose extraordinary optical properties arise from the collective oscillations of conduction electrons (plasmons) and depend critically on their geometric arrangement within an assembly [19,20]. Gold nanoparticles (AuNPs) serve as a paradigmatic example, with DNA-mediated assembly enabling their programmable organization into complex one-, two-, and three-dimensional architectures [21,22,23]. For instance, Park’s group assembled AuNPs onto triangular pyramid DNA templates via electrostatic attraction, tuning plasmonic absorption by adjusting the mixing ratio [24]. This approach for creating precise plasmonic nanoassemblies has been successfully translated into functional biosensing platforms. For example, similar DNA origami–gold nanostructure complexes have demonstrated high sensitivity in cancer theranostics [25], underscoring their significant promise for developing advanced biological sensors. The confined geometry and tunable plasmonic coupling within these structures can be exploited to enhance the detection of trace chemicals or biomolecules, potentially leading to new sensing instruments with ultra-low detection limits [20]. Furthermore, this approach has been successfully generalized to other materials, including silver and silica nanoparticles, allowing for the template-directed synthesis of nanostructures with designed geometries [26,27]. A key demonstration by Shang et al. involved the site-specific synthesis of silica nanostructures on DNA origami templates, exploiting the stronger electrostatic affinity of positively charged silica precursors for prominent double-stranded DNA regions (Figure 1a) [26].

In the field of biomedicine, DNA scaffolds are highly promising for targeted drug delivery due to their precise spatial control, programmable release kinetics, and demonstrated biocompatibility, which is evidenced by their low cytotoxicity and substantial stability in physiological environments, as reflected in the extended serum longevity and nuclease resistance exhibited by various DNA nanostructures [11]. They have been engineered to deliver diverse therapeutic agents, including the chemotherapeutic doxorubicin (DOX)—which can be loaded onto 2D/3D DNA origami and released via enzymatic digestion (Figure 1b) [28]—as well as gold nanorods and photosensitizers for photothermal and photodynamic therapies [25,29]. Innovative designs include monodisperse, programmable DNA nanospheres with editable functional arms for biosensing and therapy [30,31], icosahedral DNA nanocapsules for the spatiotemporal release of caged bioactive molecules [32], and injectable hydrogel platforms for sustained, localized gene delivery [33]. Immunostimulatory drugs can also be delivered to enhance targeting specificity [34]. Recent combinatorial strategies leverage responsive mechanisms for synergistic effects. Examples include Y-shaped DNA smart drug conjugates (sYDD) with a dual-lock release mechanism for combined chemotherapy and gene silencing [35], and DNA hydrogel-functionalized natural killer (NK) cell platforms that respond to the tumor redox microenvironment to coordinate chemo-immunotherapy [36]. A critical advantage over many inorganic carriers is their enzymatic degradability, which prevents the accumulation of cytotoxic debris and reduces long-term toxicity risks.

Capitalizing on their programmability and biocompatibility, DNA scaffolds serve as versatile platforms for constructing nanostructures that probe protein interactions and organize functional enzymatic nanodevice [37,38]. They have become valuable tools in cellular science and structural biology. Proteins such as positively charged lysozyme can be assembled via electrostatic interactions [39], while dynamic devices like DNA origami hinges allow the investigation of nucleosome mechanics [40,41]. DNA structures also serve as valuable tools in structural biology. They can act as templates for protein crystallization [42,43,44] or as fiducial markers in cryo-electron microscopy to determine structures of proteins smaller than 200 kDa [45,46]. Modular protein–DNA co-crystalline systems can further be extended to construct porous architectures for guest encapsulation [47]. When proteins of interest are loaded onto DNA nanostructures, the DNA nanostructures act as backbones to specifically control the position and number of protein molecules [48]. A significant focus is the spatial organization of enzymes to mimic natural metabolic pathways. Early work by Wilner et al. used DNA scaffolds to co-localize glucose oxidase (GOX) and horseradish peroxidase (HRP), enhancing cascade activity [49,50]. Subsequent developments include artificial hydrolase mimics that emulate cooperative catalysis [51], protective DNA nanocages that shield enzymes from protease damage [52,53], and precise spatial control exemplified by the site-specific fixation of multiple enzymes (e.g., amylase, maltase, glucokinase) on a DNA origami triangle to achieve efficient sequential cascades (Figure 1c) [54].

A key challenge in DNA scaffold design for protein science is to efficiently align proteins of interest while maintaining their activity [17]. This involves overcoming difficulties in DNA scaffold/protein interaction, such as achieving specific, high-affinity binding without interfering with the protein’s functional conformation. Strategies have employed sequence-specific DNA-binding proteins like zinc-finger proteins (ZFPs) for enzyme assembly, though challenges in affinity, efficiency, and generalizability remain [55,56,57,58]. Protein activity can be sensitive to the local environment created by the DNA scaffold, necessitating careful design to preserve enzymatic function.

Despite these challenges, successfully designed DNA scaffold origamis offer distinct advantages. DNA origami can not only coat proteins but also precisely regulate their assembly pathways, enabling the synthesis of entirely new types of protein-based materials [59]. Notably, Wang et al. circumvented the conventional reliance on long viral scaffold DNAs by employing locally interlocked short oligonucleotides, thereby overcoming limitations in structural scalability and topological complexity [60]. Overall, the primary advantages of these designed systems lie in their ability to enable precise spatial positioning on 1D, 2D, and 3D scaffolds. This precision enhances catalytic efficiency in multi-enzyme systems, allows for reversible activity regulation through conformational changes, and can improve enzyme stability by providing protective nano-environments [61,62,63]. Ongoing efforts aim to design increasingly sophisticated scaffolds that balance high assembly efficiency with superior functional performance.

### 2.2. RNA Scaffolds

RNA is a multifunctional biomolecule central to gene regulation and diverse cellular functions. Compared to the rigid DNA double helix, single-stranded RNA possesses superior flexibility, enabling it to fold into intricate three-dimensional structures. These inherent properties have fueled the rapid advancement of RNA nanotechnology. Over recent decades, the field has produced a variety of programmable RNA nanostructures that function as precise molecular scaffolds. These scaffolds can control the spatial organization and interaction of functional molecules, thereby regulating cellular processes for a wide range of nanobiotechnological applications [64]. The foundational use of RNA as a biological scaffold dates back to 2007, when Ponchon et al. developed a general method for expressing and purifying structured RNA in E. coli using a nuclease-resistant tRNA scaffold [65]. This pioneering work paved the way for subsequent structural and functional studies, leading to the gradual development of diverse RNA nanostructures as versatile biological scaffolds [66,67].

The programmability of RNA scaffolds enables precise control over the assembly of functional nanostructures for therapeutic and diagnostic purposes. RNA nanoparticles, formed via programmable self-assembly, can co-deliver multiple cargos, cross biological barriers (e.g., blood–brain, placental), and enhance delivery efficiency. Functionalization with aptamers or molecular beacons further allows for the highly sensitive, real-time detection of disease biomarkers, showcasing their potential in precision theranostics [68].

RNA scaffolds provide a versatile platform for spatially organizing enzymes and optimizing catalytic cascades, enhancing the yield of target compounds, such as alkanes [69,70] and isoprene [71]. Notably, a specific scaffold achieved a 1.43-fold increase in multienzyme efficiency [72]. They also serve as a rapid and reliable tool for synthetic translation regulation [73,74,75]. Furthermore, RNA scaffolds can also directly modulate the function of embedded RNA modules. For example, integrating the fluorescent aptamer Pepper into designed scaffolds forms aptamer-scaffold chimeras, where scaffold structure significantly influences the efficiency of aptamer folding and dye-binding activity (Figure 1e) [70]. The principle extends to dynamically regulated systems, where ligand-responsive RNA aptamers can modulate scaffold assembly and fine-tune multienzyme activity, leading to significant efficiency gains (Figure 1f) [71].

RNA scaffolds are powerful tools for precise genetic manipulation and regulation of gene expression. They can be engineered as origami templates to control the relative expression levels of enzymes in biosynthetic pathways at the translational level [76]. More directly, RNA scaffolds have been integrated into advanced gene-editing platforms. For instance, repurposing the U7 small nuclear RNA scaffold significantly enhanced the efficiency of ADAR-mediated RNA base editing, presenting a novel therapeutic avenue [77]. Similarly, CRISPR systems have been adapted using RNA scaffolds to create innovative tools for RNA proximity labeling [78] and ultra-sensitive miRNA detection [79], expanding the utility of CRISPR in RNA research and molecular diagnostics.

In summary, the studies highlighted above demonstrate the substantial potential of RNA scaffolds as programmable platforms in nanobiotechnology. They offer particular advantages for constructing complex, multifunctional nanostructures with precise spatial control, especially in contexts requiring high programmability and specific molecular recognition. Collectively, these advancements pave the way for intelligent, responsive RNA nanoplatforms that integrate multiple biological functions, thereby propelling progress in synthetic biology and targeted therapeutics. However, to fully realize this potential, a key challenge remains: the inherent instability of RNA compared to more robust scaffolds like proteins. Therefore, the future development of more stable and resilient RNA scaffolds is crucial for translating these promising laboratory innovations into robust clinical and industrial applications.

### 2.3. RNA/DNA Hybrid Scaffolds

With further understanding of DNA and RNA scaffold design, RNA/DNA composite templates have emerged as versatile platforms that combine the advantages of both nucleic acids [80,81,82]. Early work by Sturm et al. demonstrated the functional potential of such hybrids, using RNA–DNA nanostructures to design inhibitors for the ricin A chain (RTA) [80]. The assembly principles of natural RNA viruses, driven by electrostatic interactions between capsid proteins and the viral genome, provide a foundational biomimetic rationale for nucleic acid-directed assembly [81,82]. Expanding on this, Garmann’s group showed that viral RNA scaffolds hybridized with DNA could selectively nucleate capsid assembly, with the RNA segment packaged inside and the DNA hybrid protruding. This strategy enables the creation of asymmetric viral particles and facilitates their interface with DNA-functionalized surfaces or colloidal particles [83] (Figure 1d). These studies highlight a key advantage of hybrid scaffolds: they integrate the structural and functional diversity of RNA with the stability and programmable addressability of DNA, creating systems with unique properties not easily achieved by mononucleic scaffolds alone.

The programmability of RNA/DNA hybrid scaffolds has been effectively harnessed to develop highly sensitive detection systems. Palindrome-crosslinked DNA nanoclusters, which form self-assembled nanospheres, serve as a structural scaffold. By anchoring hairpin probes to create DNA–stem–hairpin (DSH) architectures, this system enables rapid and highly specific detection of circular RNA biomarkers, allowing for precise discrimination of lung cancer stages and subtypes [84]. This approach overcomes limitations of conventional assays, such as prolonged turnaround time and high cost, providing an efficient tool for early cancer screening and molecular subtyping. Furthermore, dynamic detection mechanisms can be engineered on these scaffolds. For instance, a tetrapod DNA walker constructed on a three-dimensional DNA scaffold accelerates target recognition kinetics and has been applied to the highly sensitive photoelectrochemical detection of *Phytophthora infestans* RNA [85].

In the therapeutic domain, RNA/DNA hybrid scaffolds offer promising platforms for targeted treatment strategies. Wu et al. reported a convenient method for constructing RNA/DNA hybrid origami nanostructures for effective gene therapy. The resulting nanostructures significantly inhibit tumor cell proliferation and serve as promising biomaterials for delivering therapeutic nucleic acids [86]. Beyond direct drug or gene delivery, hybrid scaffolds can also orchestrate complex biochemical reactions for therapeutic purposes. For example, protein-based programmable systems enable the spatially ordered assembly of multienzyme complexes in vitro by recruiting engineered proteins to RNA–DNA composite scaffolds via complementary guide DNAs [87]. This precise spatial control over enzymatic activity lays the groundwork for developing sophisticated synthetic metabolic pathways or diagnostic–therapeutic (theranostic) systems within a controlled nanoscale environment.

## 3. Protein Scaffolds

Although nucleic acid scaffolds offer precise spatial organization for diverse applications, their high cost and stringent operational requirements make them particularly suited for precise fields like cellular diagnostics and therapy [14,88]. In contrast, protein scaffolds serve as cost-effective and inherently biocompatible alternatives [89]. Beyond linear architectures, protein scaffolds can adopt advanced three-dimensional morphologies, such as cyclic and cage-like structures. These 3D configurations provide superior advantages over linear forms, including the creation of protective nano-environments and the enabling of higher-order spatial organization, which collectively enhance catalytic efficiency and functional integration [90,91,92,93,94,95,96,97,98].

### 3.1. Cyclic Protein

Cyclic peptides have emerged as a novel class of molecular frameworks that are highly amenable to rational design for constructing biomimetic nanostructures and functional complexes. Their programmable sequences, well-defined geometries, and capacity to engage in directional non-covalent interactions enable precise control over supramolecular assembly, offering unique opportunities to engineer materials and interfaces with tailored physicochemical and biological properties.

Protein scaffolds based on cyclic oligomers provide a versatile platform for assembling functional complexes. The rosettasome, a bicyclic chaperonin complex from the hyperthermophilic archaeon *Sulfolobus shibatae*, consists of three different subunits arranged in two nine-membered rings [99]. By fusing a cohesin module to a rosettasome subunit, engineered “roselases” capable of binding dockerin-tagged enzymes have been created. These scaffolded complexes exhibit significantly enhanced activity, achieving a 2.4-fold higher cellulose degradation rate than free enzymes [100]. Similarly, colocalization of hemicellulose-degrading enzymes on this scaffold increased product yield by 40% compared to the free enzyme mixture, demonstrating its efficacy in promoting multi-step metabolic pathways [101].

**Figure 2:**
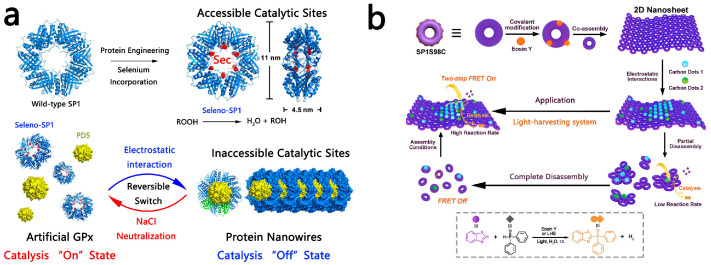
SP1 as a scaffold protein. (**a**) Design of the artificial antioxidant enzyme seleno-SP1 and reversible switching of the catalytic activity through controlled protein assembly and disassembly [107]. Copyright 2020 American Chemical Society. (**b**) Schematic representation of the construction of the light-harvesting system with two-step energy transfer process and the control of the “On/Off” state of the FRET process [110]. Copyright 2022 American Chemical Society.

Stable Protein 1 (SP1), a highly robust cyclic dodecamer from poplar, serves as an exceptionally stable and programmable scaffold [102]. Its oligomeric form exhibits remarkable tolerance to heat, proteases, pH extremes, and organic solvents. Fusion of dockerin modules to target enzymes enables their assembly on SP1, leading to a substantial increase in specific activity [103]. Liu group has systematically engineered SP1 into diverse functional nanostructures. They assembled one-dimensional SP1 nanotubes complexed with quantum dots or dendrimers via electrostatic interactions, repurposing them as artificial light-harvesting systems (LHS), bacterial energy transfer mimics, or regulable multi-enzyme antioxidant platforms (Figure 2a) [104,105,106,107]. Notable achievements include selenium-containing antioxidant systems and a nickel-responsive artificial selenoenzyme, whose activity can be reversibly modulated through assembly control [107,108]. Advancing SP1’s photonic applications, the group employed SP1-based protein nanosheets as templates to construct efficient artificial LHSs [109,110]. They developed a controllable “On/Off” switchable LHS with sequential multistep energy transfer and integrated photocatalysis (Figure 2b) [110], achieving an 80-fold enhancement in hydrogen production and redox-regulated activity switching [111]. Furthermore, integrating protein self-assembly with in situ biosynthesis yielded a highly efficient and stable biomimetic platform for photocatalytic hydrogen evolution [112]. Beyond photonics, SP1 has been used to create functional nanochannels. Gdor et al. adsorbed thiolated SP1 on gold, backfilled with sol–gel, and demonstrated charge-selective transport in the resulting nanochannel arrays [113]. Yara Zeibaq et al. conjugated a heme cofactor to SP1 to create a biohybrid with enhanced peroxidase-like activity for potential non-aqueous catalysis [114]. Kuai et al. used the SP1 cavity to template size-controlled gold clusters and further optimized their catalytic performance through mutagenesis and metal doping [115].

Other cyclic proteins also serve as effective scaffolds. Proliferating cell nuclear antigen (PCNA) from *Archaeoglobus fulgidus* is a heterotrimeric ring-shaped protein with its C-termini exposed on one face, making it an advantageous scaffold [116]. By fusing the C-terminus of each PCNA subunit with distinct enzymes involved in a cytochrome P450cam electron transfer chain (P450cam, PdX, and PdR), a stable heterotrimeric complex self-assembles. The close proximity of enzymes within this scaffold facilitates efficient electron transfer, resulting in catalytic activity two orders of magnitude higher than that of the free enzymes [117,118].

Beyond natural cyclic proteins, engineered cyclic peptide platforms have emerged. A tyrosinase-catalyzed phage display system enables one-step, high-efficiency cyclization of peptides, offering a novel strategy for developing cyclic peptide therapeutics and probes [119]. Moreover, preorganized cyclic modules guide the predictable self-assembly of complex protein nanostructures from amino acid sequences [120]. Functionalized cyclic peptide assemblies can also multivalently recognize and cluster cell surface receptors like EGFR, modulating their signaling and establishing a novel self-assembly-based paradigm for targeting membrane proteins in cancer therapy [121].

### 3.2. Protein Cages

Beyond their capacity to aggregate target proteins, inorganic nanoparticles, and therapeutic compounds, protein scaffolds demonstrate remarkable versatility by creating confined, organelle-mimetic microenvironments through cage-like architectures. These “protein cages” are defined as hollow supramolecular nanostructures composed of multiple, often identical subunits that self-assemble with precise symmetry to form well-defined cavities [122]. Structurally, they exhibit three distinct and functionally significant surfaces: an inner surface that encloses a physically isolated chamber for the encapsulation and protection of cargo molecules (e.g., bioactive compounds, enzymes); an outer surface that provides a robust platform for chemical or genetic functionalization, enabling the introduction of targeting ligands or other functional moieties; and inter-subunit interfaces that critically govern the thermodynamic stability, structural integrity, and morphological characteristics of the assembly [123]. The highly symmetric arrangement of subunits inherently generates pores at their junctions, establishing selective diffusion pathways between the encapsulated interior and the external environment, which is essential for the controlled exchange of substrates, products, and signaling molecules [124,125].

#### 3.2.1. Virus-like Particle Protein Cages

Among the most precisely engineered and structurally diverse class of protein cages are virus-like particles (VLPs). These are derived from the self-assembling capsid proteins of viruses but lack the viral genome, making them non-infectious, highly biocompatible, and programmable nanoscaffolds [126,127,128]. Their intrinsic nanoscale precision, geometric symmetry, and ability to multivalently display antigens also confer potent virus-like immunogenic properties, making them highly attractive for vaccine design [129,130]. To overcome limitations in stability, innovative engineering approaches have been employed. For instance, the introduction of an internal, covalent polymer network creates an “endoskeleton” within the VLP, significantly enhancing its structural integrity under harsh conditions such as elevated temperature or organic solvent exposure, thereby expanding its applicability [131].

**Figure 3:**
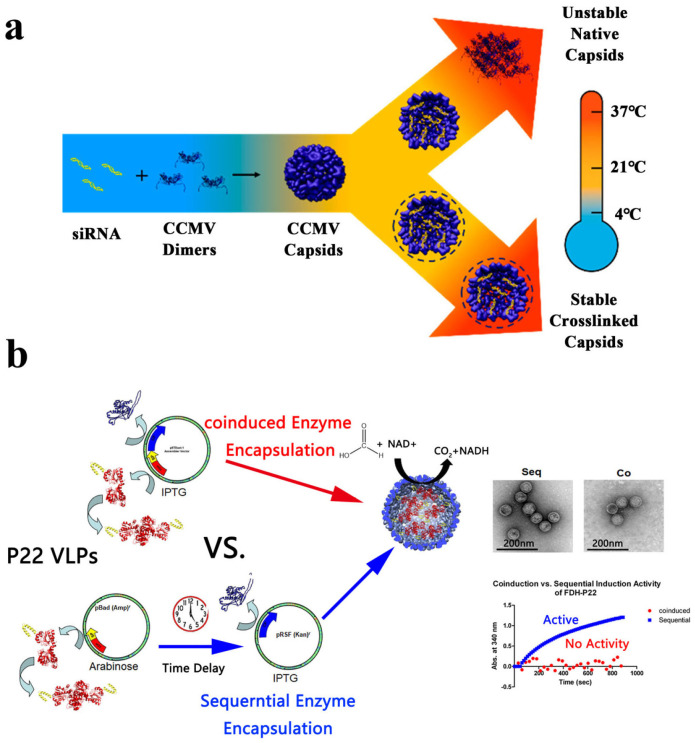
Virus-like particle protein cages as scaffold proteins. (**a**) Schematic representation of a versatile cross-linking strategy to stabilize CCMV nanoparticles after cargo loading for efficient siRNA delivery [140]. Copyright 2019 American Chemical Society. (**b**) Strategies for the encapsulation of the native formate dehydrogenase (FDH). Comparison of single-carrier and double-carrier methods [156]. Copyright 2022 American Chemical Society.

A quintessential model system is the cowpea chlorotic mottle virus (CCMV) capsid, an icosahedral assembly of 180 identical protein subunits forming a 28.6 nm cage [132]. It exhibits reversible, pH-dependent assembly (disassembling at pH 7.5 and forming at pH 5.0), a positively charged interior suitable for interaction with polyanions like RNA, and has shown excellent biocompatibility and low immunogenicity in preclinical studies, properties that can be further optimized through surface coating strategies or genetic modification approaches [133,134]. These properties have enabled multifaceted applications. For instance, CCMV VLPs serve as versatile artificial nanoreactors. One notable approach integrates them with DNA origami to achieve spatiotemporally controlled enzymatic cascades [135]. Their intrinsic protein cage structure is particularly advantageous for encapsulation. Efficient enzyme loading has been accomplished by exploiting the negative charge of nucleic acid tags for co-encapsulation at neutral pH [136], or by utilizing sortase A-mediated ligation for covalent cargo attachment [137,138]. Importantly, this covalent ligation method can likely be extended to a variety of other cargoes, for instance, catalysts or drug molecules. Beyond catalysis, CCMV VLPs have been engineered into advanced drug delivery platforms. This transition is facilitated by their unique reversible self-assembly properties and their inherent suitability for cargo loading and functionalization [139,140,141]. Key strategies include: designing reversible cross-linkers (e.g., DTSSP) to stabilize the cage during systemic circulation while allowing triggered intracellular disassembly for siRNA release (Figure 3a) [140]; and creating genetically mutated capsids capable of co-loading diagnostic agents (e.g., fluorescent dyes), targeting ligands (e.g., folic acid), and chemotherapeutic drugs (e.g., doxorubicin) for theranostic applications [142]. In a related protein engineering approach, Ki et al. designed a cage architecture with specific heparin-binding capability, enabling controlled encapsulation of biomacromolecules [143]. Recent advances further encompass dual-site selective functionalization techniques and integrated purification strategies, which pave the way for high-precision biomedical applications [144,145].

Bacteriophage-derived VLPs, particularly those from the *Salmonella typhimurium* phage P22, represent another powerful scaffold system with distinct structural and functional advantages. The P22 capsid is an icosahedral structure composed of 420 coat proteins (CP) and requires approximately 100–330 scaffold proteins (SP) for proper assembly, forming a procapsid [146]. The SP plays a crucial role not only in P22 but also in the morphogenesis of many other large viruses [147]. A key feature of P22 VLPs is their structural plasticity; they can adopt an expanded shell (EX) or a porous “wiffleball” (WB) morphology with ~10 nm openings, in addition to the procapsid form, offering tunable internal volume and porosity for diverse encapsulation needs [148]. These structural properties enable P22 VLPs to serve as versatile nanocarriers for enzyme protection and functional assembly. For example, coiled-coil-mediated assembly yields robust catalytic materials with enhanced cascade performance [149]. The system also accommodates structurally diverse guests including alkaline phosphatases [150] and chimeric proteins, providing insights into molecular crowding effects relevant to biosensing [151]. This system excels in constructing complex biocatalytic nanocompartments. Enzymes can be loaded via genetic fusion to SP or CP subunits, enabling the co-encapsulation of multi-enzyme cascades relevant to metabolism, such as those involving glucose or mevalonate [152,153,154,155]. Sophisticated assembly strategies have been developed to optimize functionality, including sortase-mediated modular attachment to the VLP exterior [148] and a sequential expression strategy that allows enzymes to fold and mature before encapsulation, thereby preserving their full activity—a solution to the common problem of inactive post-encapsulation enzymes (Figure 3b) [156]. Moreover, tunable co-encapsulation of multiple enzymes with controlled stoichiometry has been achieved in vivo, enhancing cascade efficiency [157]. Beyond biocatalysis, P22 cages have been used as nanoreactors for polymer synthesis via techniques like atom transfer radical polymerization (ATRP) initiated from the interior surface [158], and as stimuli-responsive drug delivery systems that leverage pH-responsive properties for tumor microenvironment-triggered release [159]. A groundbreaking integration has led to the creation of an “artificial photosynthetic cell factory,” where photocatalytic modules (e.g., CdS quantum dots) are coupled with enzymatic CO2 fixation machinery within the P22 cage, significantly enhancing light-driven NADH regeneration and carbon fixation efficiency [160]. In addition to P22, other bacteriophage systems such as T4 and MS2 have also been successfully employed as protein scaffolds for the immobilization of cascade enzymes, further broadening the toolkit for constructing complex biocatalytic assemblies [161,162].

The utility of VLPs as programmable protein scaffolds extends well beyond the well-characterized CCMV and P22 systems. A diverse array of other plant and bacterial viruses has been successfully engineered for nanobiotechnology applications. For instance, the pseudofilamentous Potato Virus X (PVX) has been genetically modified to display enzymes like Candida antarctica lipase B (CALB) on its coat protein, creating a biocatalytic scaffold [126]. The efficiency of such assemblies can be further enhanced using robust conjugation technologies; the SpyTag/SpyCatcher system has been employed to covalently attach an endoglucanase (Cel12A) to PVX nanoparticles, resulting in improved substrate conversion compared to the free enzyme [163]. In the realm of therapeutics, the Brome Mosaic Virus (BMV) has demonstrated significant potential. Its capsid has been effectively loaded with small interfering RNA (siRNA), and subsequent delivery to tumor cells resulted in cargo release and successful gene silencing, all while exhibiting a low immunogenic profile—a highly desirable combination for molecular therapy [164]. These examples underscore the broader principle that the highly modular and designable nature of VLPs, combined with advanced bio-conjugation and encapsulation techniques, establishes them as a versatile and powerful platform. They are capable of addressing complex challenges across biocatalysis, biosensing, targeted drug delivery, and gene therapy by providing spatially organized, protective, and functionalizable nanocompartments [163,164,165].

#### 3.2.2. Non-Viral Protein Cages

Beyond the extensively characterized viral capsids, a diverse array of naturally occurring, non-viral protein cages offers a complementary and versatile toolkit for nanotechnology. These biomolecular architectures, derived from organisms across the biological spectrum, exhibit a wide range of sizes, shapes, chemical stabilities, and assembly properties, making them highly attractive as programmable scaffolds [166]. Their intrinsic biocompatibility and structural precision enable applications ranging from targeted therapeutic delivery and bioimaging to biocatalysis and synthetic biology. This section highlights key representatives of these natural nanocages—particularly ferritin and lumazine synthase—and briefly introduces emerging artificial designs.

**Figure 4:**
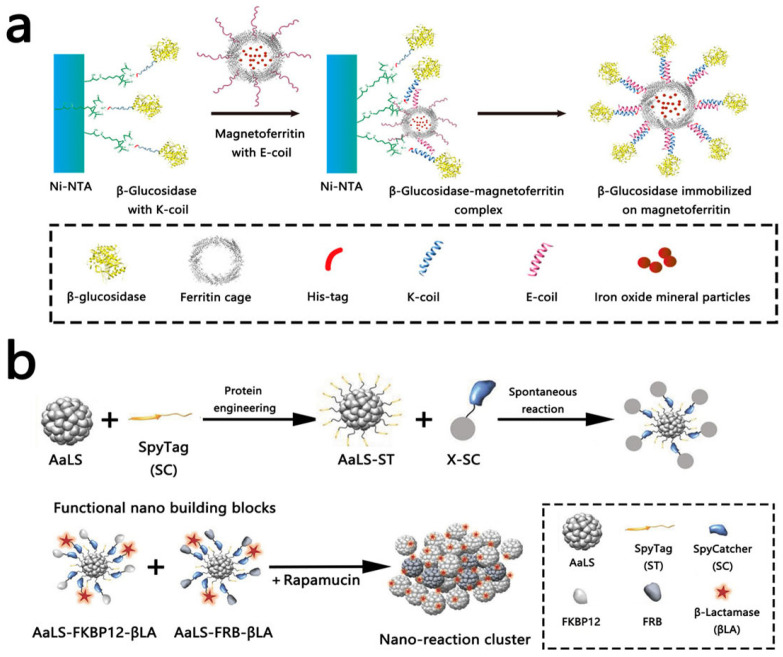
Non-viral protein cages as scaffold proteins. (**a**) Schematic of purification of β-glucosidase and the human H chain ferritin without the sequence of a His-tag (KG-HE) protein complex [192]. Copyright 2019 MDPI. (**b**) Schematic representation of AaLS protein cage as nanobuilding blocks and their cluster formation [200]. Copyright 2018 Wiley.

One of the most well-studied and versatile natural nanocages is Ferritin, a ubiquitously expressed iron-storage protein that self-assembles from 24 identical subunits into a robust, spherical cage (outer diameter ~12 nm, inner cavity ~8 nm). It exhibits exceptional stability across a broad temperature (4 °C to 80 °C) and pH (2 to 11) range, which underpins its utility as a reliable scaffold [167,168,169]. In biotherapeutics, ferritin has been engineered for the targeted delivery of various anticancer agents (e.g., doxorubicin, paclitaxel, cisplatin, PROTACs), enhancing tumor-specific accumulation and efficacy while reducing systemic toxicity [170,171,172,173]. Its surface can be functionalized to co-deliver targeting peptides, siRNA, or enzymes, enabling synergistic combination therapies [174,175,176]. Applications extend to neurological disorders, such as co-encapsulating metformin and rapamycin for autism spectrum disorder intervention [177], and to oral peptide delivery, where it protects therapeutics like GLP-1 from gastrointestinal degradation [178]. Structural insights from cryo-EM reveal its role as a template for soft metal nanoclusters [179] and the diversity of its channel architectures, guiding engineering efforts [180]. In diagnostics and imaging, ferritin serves as a carrier for fluorescent probes (e.g., IR820, Cy5.5) and theranostic agents like verteporfin [181,182,183,184]. Its capacity for high-density, ordered antigen display also makes it a potent vaccine platform, with candidates against influenza, SARS-CoV-2, and Epstein–Barr virus in clinical development [168,185,186,187,188]. In biocatalysis, ferritin’s negatively charged interior facilitates enzyme encapsulation (e.g., carbonic anhydrase) via electrostatic interactions [189,190], while strategic mutagenesis enables the creation of artificial metalloenzymes [190,191]. Its intrinsic or engineered magnetic properties further allow the construction of recoverable biocatalysts (Figure 4a) [192,193].

Another exemplary natural system is Lumazine Synthase (AaLS), originally from the hyperthermophile *Aquifex aeolicus*. It forms a thermostable, hollow icosahedron of 60 subunits (outer diameter ~15 nm, inner diameter ~9 nm) [194,195]. Its robust, negatively charged cage is ideal for cargo encapsulation via electrostatic interactions, with studies demonstrating the encapsulation of various guest proteins and the influence of ionic strength on assembly, sometimes yielding complex multi-shell structures [196,197,198,199]. Surface functionalization strategies, such as covalent display of enzymes (e.g., β-lactamase) and ligands, enable the creation of bioactive building blocks. Subsequent layer-by-layer assembly into nanoclusters can amplify enzymatic activity (Figure 4b) [200]. Beyond encapsulation, AaLS serves as a scaffold for nanomaterial synthesis, antigen delivery, targeted drug delivery, and nucleic acid encapsulation [201,202,203]. In vaccine development, AaLS-based platforms conjugated with antigens from pathogens like SARS-CoV-2 and dengue virus have elicited potent immune responses [204,205,206]. Advanced designs include mosaic nanoparticles for broad-spectrum immunity [207,208] and thermostable formulations [209]. In oncology, AaLS-derived systems have been engineered as bifunctional nanotherapeutics to combat multidrug resistance [210] or to modulate the tumor microenvironment via enzyme delivery [211]. Mechanistic studies on its assembly dynamics support the rational design of multifunctional platforms [206].

The repertoire of useful non-viral cages extends to other natural systems such as DNA-binding proteins from starved cells (Dps), heat shock proteins, chaperonins (e.g., thermosomes used as ATRP nanoreactors), and metabolic complexes [212,213,214]. Furthermore, advances in computational protein design have enabled the de novo creation of artificial protein cages with novel geometries and properties. By redesigning the interfaces of protein building blocks, researchers have induced self-assembly into symmetric cages not observed in nature [215,216,217]. Several designed systems, including O3-33, I53-50, and I3-01, have shown functional promise [166,217,218,219,220,221]. For instance, the octahedral cage O3-33 (24 subunits, ~13 nm diameter) can be produced in E. coli and engineered for nucleic acid delivery by imparting a positive charge to its lumen for siRNA binding and cellular delivery [222]. It also finds use in biocatalysis, such as when fused to an enzyme to enhance bioelectrode stability [223]. Controllable assembly methods, like protease-triggered cage formation, allow precise encapsulation of diverse cargoes (proteins, dyes, drugs) for applications in delivery and nanoreactors [224]. While the field is progressing rapidly, future efforts aim to design cages with larger cavities, enhanced stability, simpler production, and greater modularity to fully realize their potential as next-generation programmable biomaterials.

### 3.3. Other Protein Scaffolds

Beyond the well-defined cage and cyclic protein assemblies, several other protein systems possess an inherent capacity to form functional scaffolds. Notable examples include bacterial microcompartment shell proteins, amyloid fibrils, and casein micelles, each offering unique structural and functional properties for nanobiotechnology applications [220,225,226,227,228].

Bacterial microcompartments are prokaryotic organelles comprising a semi-permeable protein shell that encapsulates specific metabolic enzymes. Individual shell proteins can self-associate into higher-order structures, forming the basis for engineered scaffolds [229]. Synthetic BMC shells, built from proteins containing Pfam00936 or Pfam03319 domains, function as nano-factories to encapsulate compounds for biosynthesis [230]. For instance, Schmidt-Dannert’s group leveraged the self-assembly of EutM shell proteins from *Salmonella enterica* to create hybrid scaffolds with integrated enzyme attachment sites [231]. By employing SpyTag-SpyCatcher covalent chemistry, they constructed biocatalyst–scaffold conjugates that significantly reduced reaction times compared to free enzyme systems [232]. Similarly, Chen et al. co-immobilized α-amylase and trehalose synthase on EutM scaffolds, creating a cascade system with enhanced activity and stability for efficient trehalose production [233]. These works illustrate the potential of repurposed BMC proteins as modular scaffolds for spatially organizing enzymes [234].

Amyloid fibrils represent another class of protein-based scaffolds. Traditionally associated with disease, their exceptional stability, high aspect ratio, and capacity for functionalization have been exploited in material science. Engineered amyloid-like peptides can self-assemble into nanowires, hydrogels, or films that serve as templates for nanoparticle organization or as supportive matrices for cell culture and tissue engineering. Their surface can be chemically modified or genetically fused with functional motifs, enabling the precise display of catalysts, ligands, or drugs, thus offering a versatile platform for catalytic nanostructures and bioactive materials.

Casein micelles, the natural protein assemblies found in milk, are inherently biocompatible and nutritive nanocarriers. Their porous, sponge-like structure and capacity for hydrophobic interaction make them excellent scaffolds for encapsulating and delivering bioactive compounds, such as vitamins, antioxidants, and hydrophobic drugs. Researchers have utilized casein micelles and their individual components (e.g., β-casein) to improve the solubility, stability, and controlled release of nutraceuticals and pharmaceuticals in food and biomedical applications, highlighting their role as natural, food-grade delivery scaffolds.

Artificial-intelligence-driven protein design frameworks have enabled the efficient mining of novel scaffolds with synthetic binding protein characteristics from the entire protein sequence space, establishing a new paradigm for antibody-alternative therapeutics and high-specificity diagnostic reagents [235]. Concurrently, the development of multiple high-throughput scaffold screening strategies has significantly enhanced the targeting precision and cargo-loading efficiency of engineered extracellular vesicles (EVs) in drug delivery and targeted therapy, providing critical technological support for the clinical translation of EV-based therapeutics [235,236,237,238].

Taken together, these diverse protein systems—encompassing bacterial microcompartments, amyloid fibrils, and casein micelles—alongside the emerging technologies of computational design and high-throughput screening, provide a broad and powerful toolkit for developing next-generation scaffolds with tailored functions for biocatalysis and therapeutic applications.

## 4. Conclusions and Perspectives

In this review, we summarize nucleic acid and protein scaffolds, and analyze representative systems across different classes of biological nanoscaffolds. These systems, along with their key characteristics and primary application advantages, are comparatively summarized (Table 1). We found that each backbone has different characteristics due to the diversity of building blocks, which allows us to rationalize the selection and design of suitable biological scaffolds as needed.

Biological nanoscaffolds, despite their remarkable programmability and versatility, face significant translational hurdles that span from fundamental fabrication to clinical application. A primary challenge lies in their synthesis and production, where achieving cost-effective, scalable, and reproducible manufacturing of complex nanostructures remains difficult for both nucleic acid and protein systems. Beyond assembly, the functionalization and cargo loading processes necessary to imbue these scaffolds with therapeutic or diagnostic functions often involve intricate chemistries that can compromise the activity of the cargo or the structural integrity of the scaffold itself. Furthermore, ensuring sufficient stability in biological environments is critical for in vivo applications, as scaffolds must be engineered to resist enzymatic degradation, premature immune clearance, and destabilization under physiological conditions. Finally, the path from the laboratory bench to the clinic is governed by regulatory and translational aspects, which include the need for standardized characterization protocols, comprehensive long-term safety and biodistribution studies, and navigation of complex regulatory pathways for novel biomaterials. Addressing these interconnected challenges is essential for unlocking the full practical potential of biological nanoscaffolds.

Beyond these technical and regulatory hurdles, proactive safety assurance is paramount for the clinical translation and public acceptance of biological nanoscaffolds. It is essential to directly address legitimate concerns regarding potential risks, such as the theoretical possibility of engineered protein scaffolds inducing aberrant aggregation (prion-like behavior) or of nucleic acid scaffolds integrating into the host genome and causing insertional mutagenesis. The field is actively developing comprehensive safety-by-design strategies to mitigate these risks. For protein scaffolds, this includes rigorous selection of non-pathogenic, highly stable parental folds, incorporation of destabilizing elements to prevent off-pathway aggregation, and implementation of sensitive in vitro and in vivo assays to screen for any amyloidogenic or immunotoxic tendencies. For nucleic acid scaffolds, strategies focus on using non-integrating vectors, incorporating chemically modified nucleotides to enhance stability and reduce immune stimulation, and designing self-limiting or biodegradable structures that degrade after fulfilling their function. Establishing standardized, long-term toxicological and biodistribution profiles for each new scaffold platform will be critical. Ultimately, transparent communication of these risk-assessment frameworks and safety data, alongside continued dialogue between scientists, clinicians, and regulators, will be key to building the necessary trust for these transformative technologies to realize their therapeutic potential.

The research on nucleic acid scaffolds is gradually deepening with the development of nucleic acid nanotechnologies and computer technologies. With computational design, more complex structures can be fabricated for the construction of nanoscale bioscaffolds. However, the design and construction of nucleic acid backbones require higher costs and more stringent operational requirements than protein backbones, so they are better suited to medical applications demanding high precision and reliability. Protein scaffolds, including cyclic, cage-shaped, and other complex structures of protein assemblies, exhibit structural and functional diversity due to the differences in the morphology and properties of their building blocks. Naturally occurring self-assembling proteins from plants, animals, bacteria, and viruses serve as promising sources for scaffold development and have already been harnessed in various bioengineering applications. In addition, through precise genetic engineering and modification, natural proteins can be targeted and modified to build intelligent, editable protein nanoscaffold systems for biological and medical applications as desired. For protein scaffolds, the main problems are: how to heterologously express natural proteins and artificially designed proteins efficiently; how to ensure accurate assembly into the designed structures while maintaining functional activity; and how to selectively load or transport functional molecules or particles through rational scaffold design. Mutation technologies provide effective ideas to solve these potential problems and build more stable and controllable scaffold structures. In addition, the unique biocompatibility of artificial biological scaffolds offers the possibility of further intelligence (e.g., stimulus-responsive functionality) and functioning in living organisms.

In conclusion, biological scaffold systems, whether nucleic acid scaffolds or protein scaffolds, are highly promising core architectures. With the development of structural design and preparation technology for biological scaffolds, more efficient and intelligent three-dimensional scaffold systems are expected to be available in the future to meet the needs of applications. It is hoped that this review can provide useful insights and potential directions for the further development of nucleic acid- and protein-inspired biological nanoscaffolds, as outlined and compared herein, thereby encouraging innovative research in this evolving field.

## Figures and Tables

**Table 1:** Applications of biological scaffolds in inorganic nanoparticles assembly, enzymes assembly, and drug delivery.

Applications	Building Blocks	Structures	Advantages	References	Platform Selection Guidance
Assemble inorganic nanostructures	DNA	ssDNA scaffold	Editable, precision, and programmability	[21]	Precise spatial control needed
		DNA origami cage		[22]	Nanoparticle encapsulation
		DNA origami and ds DNA		[24,28]	Complex 3D architectures
		DNA origami tube		[27]	Tubular or wireframe scaffolds
Drug delivery	DNA	ssDNA scaffold	Biocompatibility, low cytotoxicity, high targeting and programmed for controlled release	[32]	Low toxicity, biocompatible delivery
		DNA origami triangle or tube		[25,28,29]	Stimuli-responsive release
		DNA nanostructure		[30,32]	Multifunctional carriers theranostic platforms
	DNA/RNA	Origami structure		[7]	Biosensor
	CCMV	Nanocage	Enhance the stability, sustained release of drug, easy to assemble	[139,140,141,142,143]	Long-term stability, biodegradable
	Ferritin			[170,171,172,173,174,175,176,177,178,179,180]	Iron/metalloenzyme delivery
	AaLS			[202,209,210,211]	Hydrophobic cargo in water
	O3-33			[222]	High loading efficiency
Assembly enzyme	DNA	ssDNA scaffold	Enhance the cascade activity, spatial modulation, programmable	[49,50,56,61]	Multi-enzyme pathway organization
		DNA origami cage		[53]	Enzyme protection/isolation
		DNA origami triangle		[54]	Directional enzyme placement
	RNA	RNA origami		[69,70,71,72,76]	Transient expression systems
	Rosettasomes	Cyclic protein	Explicit structure, easy to assemble	[100,101]	Scalable modular assemblies
	SP1			[103]	Confined-space kinetics
	PCNA			[117,118]	DNA repair-related networks
	CCMV	Nanocage	Improve enzyme stability, high load capacity, enhance the cascade activity	[136,138]	Stabilize labile enzymes
	P22			[148,152,153,154,155,156]	Co-localize enzymes
	Ferritin			[189,190,191,192]	Iron-dependent enzymes/antioxidants
	AaLS			[200]	Enzyme immobilization
	O3-33			[223]	Cascade reactions

## Data Availability

Not applicable.
